# Impact of Changes to National Hypertension Guidelines on Hypertension Management and Outcomes in the United Kingdom

**DOI:** 10.1161/HYPERTENSIONAHA.119.13926

**Published:** 2019-12-23

**Authors:** Sarah L. Lay-Flurrie, James P. Sheppard, Richard J. Stevens, Christian Mallen, Carl Heneghan, F.D. Richard Hobbs, Bryan Williams, Jonathan Mant, Richard J. McManus

**Affiliations:** 1 From the Nuffield Department of Primary Care Health Sciences, University of Oxford, Oxford, UK (S.L.L.-F., J.P.S., R.J.S., C.H., F.D.R.H., R.J.M.); 2 School for Primary, Community and Social Care, Keele University, Keele, UK (C.M.); 3 Institute of Cardiovascular Science, University College London, London, UK (B.W.); 4 Department of Public Health and Primary Care, University of Cambridge, Cambridge, UK (J.M.).

**Keywords:** blood pressure, general practice, hypertension, myocardial infarction, practice guideline

## Abstract

In recent years, national and international guidelines have recommended the use of out-of-office blood pressure monitoring for diagnosing hypertension. Despite evidence of cost-effectiveness, critics expressed concerns this would increase cardiovascular morbidity. We assessed the impact of these changes on the incidence of hypertension, out-of-office monitoring and cardiovascular morbidity using routine clinical data from English general practices, linked to inpatient hospital, mortality, and socio-economic status data. We studied 3 937 191 adults with median follow-up of 4.2 years (49% men, mean age=39.7 years) between April 1, 2006 and March 31, 2017. Interrupted time series analysis was used to examine the impact of changes to English hypertension guidelines in 2011 on incidence of hypertension (primary outcome). Secondary outcomes included rate of out-of-office monitoring and cardiovascular events. Across the study period, incidence of hypertension fell from 2.1 to 1.4 per 100 person-years. The change in guidance in 2011 was not associated with an immediate change in incidence (change in rate=0.01 [95% CI, −0.18–0.20]) but did result in a leveling out of the downward trend (change in yearly trend =0.09 [95% CI, 0.04–0.15]). Ambulatory monitoring increased significantly in 2011/2012 (change in rate =0.52 [95% CI, 0.43–0.60]). The rate of cardiovascular events remained unchanged (change in rate =−0.02 [95% CI, −0.05–0.02]). In summary, changes to hypertension guidelines in 2011 were associated with a stabilisation in incidence and no increase in cardiovascular events. Guidelines should continue to recommend out-of-office monitoring for diagnosis of hypertension.

## Abstract 2

Supplemental Digital Content is available in the text.

High blood pressure is a leading risk factor for stroke and coronary heart disease and subsequent disability internationally.^1^ A 10 mm Hg reduction in blood pressure (BP) is estimated to lead to a 41% reduction in stroke and a 22% reduction in coronary heart disease.^2^ Lowering blood pressure with pharmacological and nonpharmacological methods is both clinically and cost-effective.^3^ Ensuring that the correct people receive such treatment maximizes benefit whilst reducing adverse effects.

The 2011 National Institute for Health and Care Excellence (NICE) Hypertension Guideline introduced, for the first time in the United Kingdom, recommendations for the diagnosis of hypertension that included the use of out-of-office measurement for confirmation of an initially raised clinic blood pressure.^3^ The routine use of out-of-office monitoring in the diagnosis of hypertension is now also recommended in Canada, the United States, Japan, and Europe.^4–7^ This change was in response to concerns that using clinic blood pressure may result in ≈25% of individuals being misclassified due to white coat hypertension, leading to potential unnecessary costs and adverse events.^8^ Furthermore, the use of ambulatory blood pressure monitoring (ABPM), and to a lesser extent home blood pressure monitoring, reduces this misclassification^8^ and is cost-effective compared with clinic monitoring.^9^

However, these changes were not without controversy. It was suggested that implementation would result in an additional 5000 cardiovascular events each year in England and Wales.^10^ Others argued that the use of ambulatory monitoring in the diagnosis of hypertension was inappropriate given that most evidence related to the treatment of hypertension is based on clinic blood pressure readings.^11^ Conversely, at a time of increasing workload and decreasing funding in UK General Practice,^12,13^ the changes were predicted to reduce clinical workload due to a reduction in the unnecessary treatment of white coat hypertension.^3^

Sufficient time has now passed since the introduction of the NICE 2011 guideline to assess its impact on the management of hypertension and outcomes. We aimed to use data from the Clinical Practice Research Datalink (CPRD), a large database of routine electronic health records from primary care in England, to examine trends in the incidence of hypertension, use of out-of-office blood pressure monitoring, and cardiovascular morbidity and mortality from 2006 to 2017. We further aimed to test whether the introduction of the revised NICE Hypertension guideline in 2011 was associated with a change in these trends.

## Methods

The data used in this study were obtained under licence from the Medicines and Healthcare products Regulatory Agency, which does not permit data sharing. Equivalent data may be obtained subject to the terms outlined here: https://www.cprd.com/research-applications.

### Study Design

This was a retrospective cohort study of adults (aged 18 years and over) registered at general practices contributing to the CPRD between April 1, 2006 and March 31, 2017. The CPRD is a research database of routinely collected primary care records, drawn from over 600 general practices and 11 million patients who are representative of the UK population.^14^ Data from CPRD were linked to inpatient Hospital Episodes Statistics (hospital data), Office for National Statistics mortality register data, and Index of Multiple Deprivation data (describing patient socio-economic status). Patients were eligible for inclusion if their records met basic quality measures, such as nontemporary registration and practice registration occurring after birth (termed acceptable records by CPRD) and were eligible for data linkage (which meant patients registered to English practices only). Furthermore, patients had to be registered at practices with continuous data reporting during the study period (termed up-to-standard by CPRD). Patients were excluded if they had a history of hypertension before study entry, because the change in guidance was limited to the use of out-of-office monitoring for diagnosis alone. We hypothesized that the change in guidance would, therefore, have a limited impact on those with a long-standing diagnosis of hypertension. See the extended methods in the online-only Data Supplement for further details and sample size calculations.^15,16^ The study protocol was approved by the Independent Scientific Advisory Committee to CPRD (protocol number 17_239R) and was made available during peer review.

### Outcomes

The primary outcome was incidence of hypertension (defined as the presence of a first diagnostic code in the primary care record; Table S1 in the online-only Data Supplement). We considered the incidence of treated hypertension (presence of a clinical diagnostic code with antihypertensive treatment within 30 days) and untreated hypertension (presence of a diagnostic code without treatment). In sensitivity analyses, incidence of hypertension was defined according to (1) a clinical code for hypertension with or without a subsequent record of antihypertensive treatment or (2) 2 raised BP readings (clinic BP ≥140/90 mm Hg or out-of-office BP ≥135/85 mm Hg) followed by a record of antihypertensive treatment.

We studied the following prespecified secondary outcomes: rate of new prescriptions of antihypertensive medication, rate of blood pressure monitoring (clinic, ambulatory, home, and overall), incidence of (major) cardiovascular events (cardiovascular death, myocardial infarction, or stroke), cardiovascular mortality, and all-cause mortality. The specific definitions for each outcome are given in the extended methods in the online-only Data Supplement.

### Statistical Analysis

For each outcome, rates per 100 person-years were calculated for each age-sex stratum (male/female and 18–24, 25–44, 45–54, 55–64, 65–74, 75–84, and 85+) in each month. In primary analyses, rates were standardized to the English National Population standard in 2015 to account for changes in the age-sex distribution over time. In sensitivity analyses, rates were standardized according to the age, sex and socio-economic status (Index of Multiple Deprivation) of the cohort in March 2017. We performed prespecified subgroup analysis in those who had/had not developed hypertension before the beginning of each month from April 2007 onwards (allowing 1 year for incident hypertension cases to develop).

Standardized rates were examined using interrupted time series analysis.^17^ Interrupted time series analysis is appropriate for examining the impact of health interventions and policies at a population level, when such interventions have been implemented at a clearly defined point in time.^17^ We assessed whether the introduction of the NICE Hypertension guideline in 2011 was associated with a step change in rates or a change in trend, by interrupting the time series between April 1, 2011 and March 31, 2012. Since patients could enter and exit the cohort at varying time points across the entire study period, monthly rates were calculated using data from varying person-years of observation. To account for this, analytical weights (equal to the person-years of observation) were used to estimate weighted interrupted time series models. We investigated the presence of autocorrelation and the influence of seasonality by including lag terms (up to order 12) in sensitivity analyses.

In post hoc analyses, we examined the incidence of a control condition, asthma. Asthma (like hypertension) is diagnosed, monitored, and managed in primary care but (unlike hypertension) should not be causally related to changes in hypertension guidelines. Inclusion of a negative control helps ensure that any observed changes in outcomes are plausibly related to the exposure of interest rather than secular trends.^18^ We also examined the incidence of hypertension and major cardiovascular events in practices with high rates of reported out-of-office monitoring (top 20% between 2006 and 2017) to assess the possible impact of coding differences.

Data on age and sex were complete. Patients with missing deprivation data (0.08%) were assumed to be in the middle quintile of deprivation. For all outcomes, absence of a relevant diagnostic or medication code was assumed to reflect absence of disease/treatment. All analysis was conducted using Stata version 14.^19^ Further details of data cleaning and analysis are given in the extended methods section in the online-only Data Supplement. S.L. Lay-Flurrie had access to the full CPRD database and conducted all data cleaning and analysis. Linked data was provided directly by CPRD.

## Results

**Figure 1.**
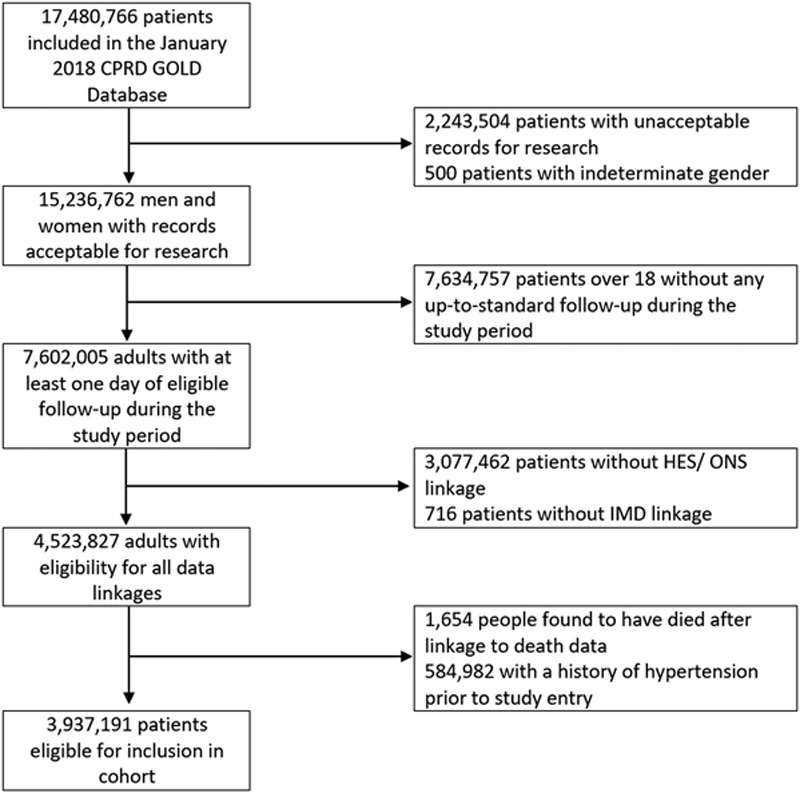
Study flow-chart. Note: Records are termed acceptable if they meet basic quality measures. Practices reporting data continuously during the study period are termed up-to-standard. CPRD indicates Clinical Practice Research Datalink; HES, Hospital Episode Statistics; and ONS, Office for National Statistics.

In total 3 937 191 patients from the January 2018 CPRD database were eligible for inclusion in the study cohort (Figure 1). Mean age at study entry was 39.7 years (SD=17.3), and 49.0% were male. There were 19 088 414 person-years of follow-up in total, and median follow-up was 4.2 years (interquartile range, 1.6–8.0). The characteristics of the cohort at study entry are given in Table 1.

**Table 1.**
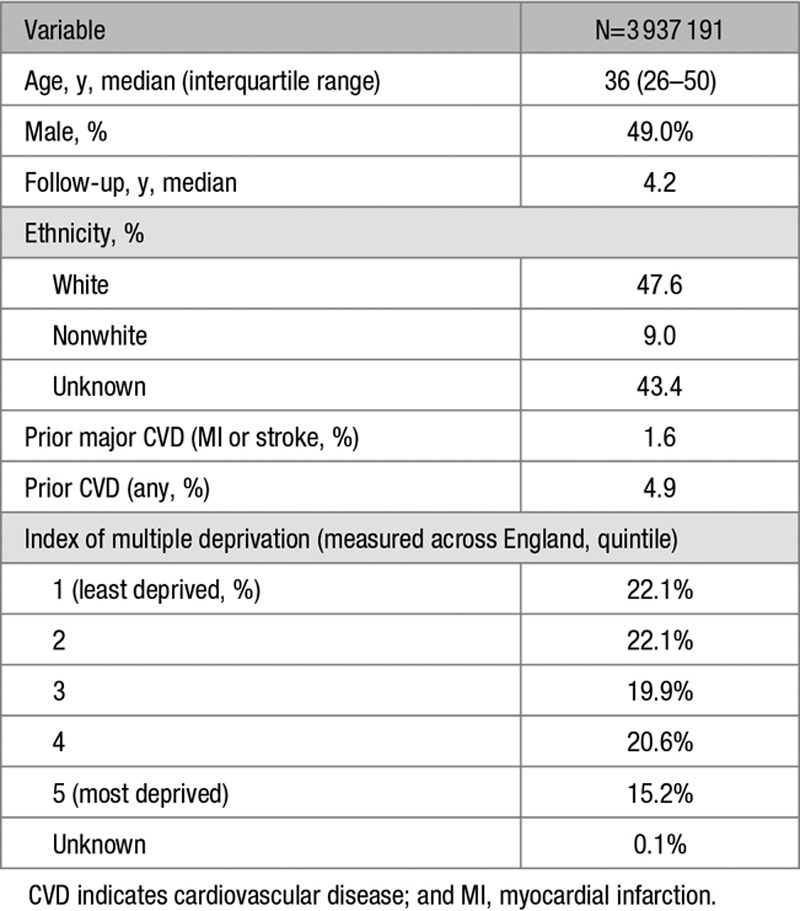
Baseline Study Characteristics

### Incidence of Hypertension

**Figure 2.**
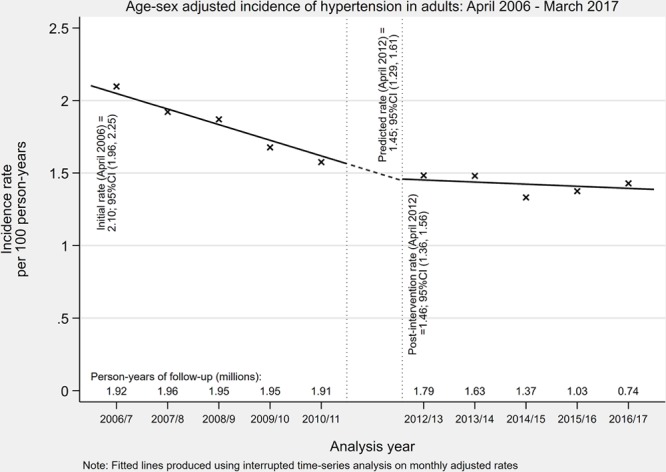
Age and sex standardized incident rate of hypertension (per 100 person-years) between April 2006 and March 2017, with interruption between April 2011 and March 2012.

In total, 236 554 (6.0%) patients developed incident hypertension during the study period (crude incidence rate of 1.31 per 100 person-years; Table S2). The age and sex standardized incidence of hypertension decreased over the study period from 2.10 per 100 person-years (95% CI, 1.96–2.25) in April 2006 to 1.39 per 100 person-years (95% CI, 1.28–1.49) in March 2017 (Figure 2 and Table 2). The year 2011, when the revised NICE hypertension guideline was introduced, was not associated with a significant change in the incident rate level (change in rate =0.01 [95 % CI, −0.18–0.20]). It was, however, associated with a reduction in the yearly downward trend (change in trend =0.093 [95% CI, 0.035–0.151]), but a similar reduction was observed in the negative control condition, asthma (Figure S1). The majority of incident hypertension cases were treated, and the same pattern in incidence was observed in treated and untreated groups (Table S3). When considering a more sensitive definition of hypertension, the incidence rate was higher but patterns in adjusted rates were similar (Table S4).

**Table 2.**
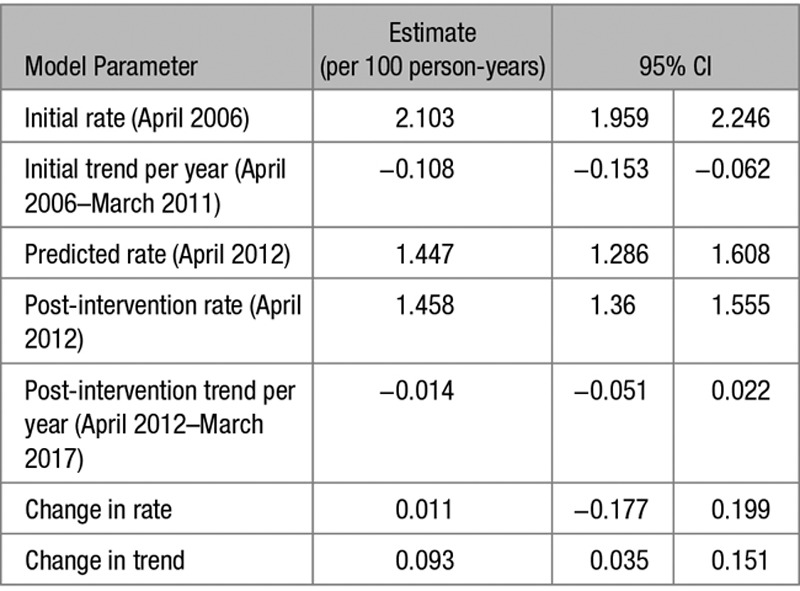
Results From Interrupted Time Series Analysis of Age and Sex Standardized Rates of Incident Hypertension Between April 2006 and March 2017, With Interruption Between April 2011 and March 2012

### Blood Pressure Monitoring

**Figure 3.**
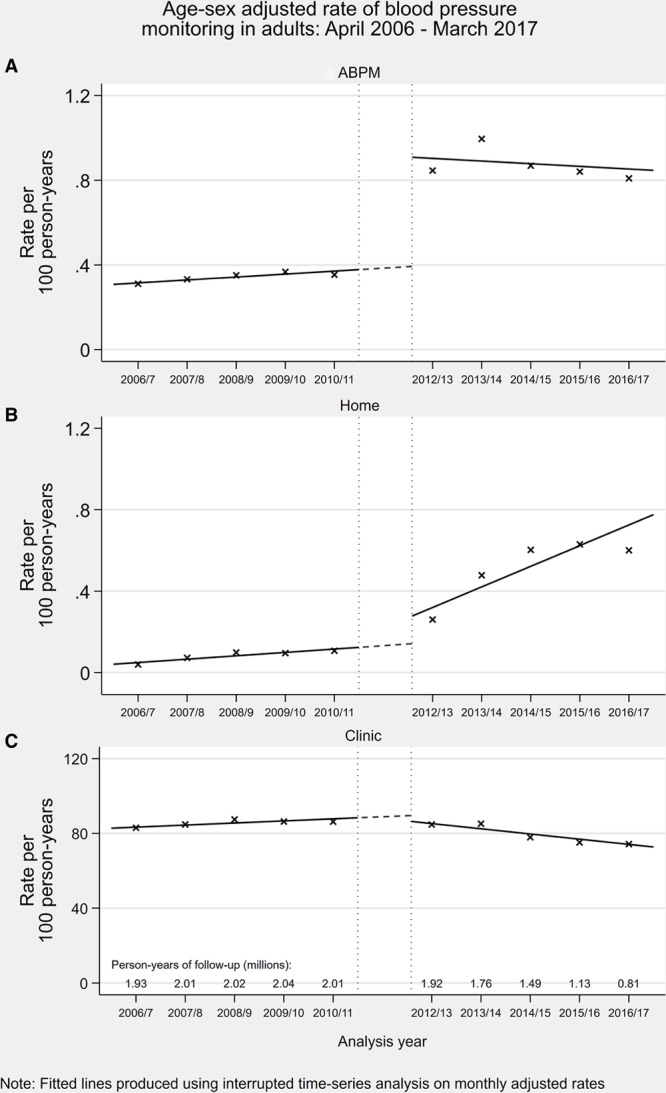
Age and sex standardized rate of blood pressure monitoring (per 100 person-years) by month from April 2006 to March 2017 with interruption between April 2011 and March 2012.

Patients were recorded as having home and ambulatory monitoring on 39 260 and 98 071 occasions, respectively (crude rate of 0.2 and 0.5 per 100 person-years, respectively). In comparison, patients had recorded clinic blood pressure on 14 717 205 occasions (crude rate of 77.1 per 100 person-years). The change in guidance in 2011 was associated with a significant change in the age and sex standardized rates of home and ABPM and a significant change in the trend for home monitoring (Table S5 and Figure 3). The 2011 guidance was associated with a change in a downwards trend of clinic BP monitoring but not a change in the level. In those with a diagnosis of hypertension, the proportion who had a record of home or ambulatory BP monitoring before their diagnosis increased from 3.7% for those diagnosed in 2006/2007 to 25.6% in 2016/2017.

### Cardiovascular Morbidity, Mortality, and All-Cause Mortality

**Figure 4.**
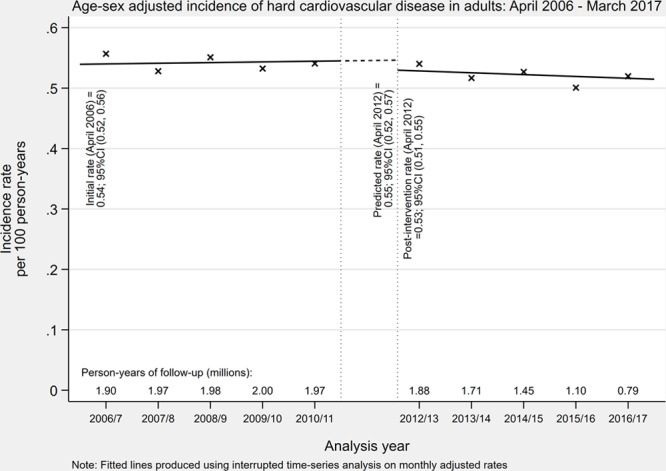
Age and sex standardized incident rate of major cardiovascular disease (per 100 person-years) between April 2006 and March 2017 with interruption between April 2011 and March 2012.

After excluding 63 623 patients with a history of major cardiovascular disease (myocardial infarction or stroke) before study entry, 66 785 patients (1.7%) had an incident MI or stroke during the study period (crude incidence rate of 0.36 per 100 person-years). The standardized incidence rate of cardiovascular disease was unchanged across the study period (Figure 4). The introduction of new NICE guidance in 2011 was not associated with changes in the incidence rate or trend in CVD (Table S6). Rates of any CVD and all-cause mortality were unchanged across the study period, but there were small (borderline significant) changes in trend for rates of major CVD mortality and any CVD mortality (5–8/100 000 patient years per year change).

### New Antihypertensive Medication Use

In total 11% of patients (399008 of 3 643 319) received antihypertensive medications for the first time during the study period (crude incidence rate of 2.5 per 100 person-years). The age-sex standardized rate of new antihypertensive medication use decreased during the study period (Figure S2 and Table S7). The change in guidance in 2011 was not associated with a change in rate (change in rate =−0.105 [95% CI, −0.406 to 0.196]) but was associated with a flattening of the previously downward trend (change in trend =0.096 [95% CI, 0.007–0.185]). There was no clear change in the pattern of prescribing of at least one medication in each class (Figure S3).

### Sensitivity and Post Hoc Analyses

There was some evidence of seasonality in rates but accounting for this produced similar results to primary analyses. An exception was for our broad definition of (any) CVD events, where the change in guidance was associated with a small decrease in the incidence rate level and a flattening trend (data not shown). Results for incidence of hypertension were also similar when standardizing according to age, sex, and deprivation in the final month of analysis (data not shown).

Results stratified by hypertensive status are given in Figures S4 through S8 and Table S8. Absolute rates of BP monitoring, incident CVD, and incident antihypertensive treatment were higher in patients with hypertension compared with those without. The change in guidance in 2011 was associated with larger absolute changes in the rate of ambulatory blood pressure monitoring and home monitoring in hypertensive compared with normotensive groups but similar relative changes. Yearly trends in the rate of ambulatory and home monitoring remained largely flat across the study period in normotensive patients. Conversely, in hypertensive patients, trends in the rate of ambulatory and home BP monitoring changed significantly (from decreasing to flat and flat to increasing respectively). There was little change in the rate of office BP monitoring which continued its downward trend. For incident major CVD events (myocardial infarction and stroke), rates remained unchanged in both hypertensive and normotensive groups. Finally, the change in guidance was associated with a large increase in the rate of new antihypertensive treatment in patients with hypertension but no change in patients without.

In practices with high rates of recorded out-of-office monitoring, the incidence of hypertension and major CVD events were similar to that observed in all practices (Figures S9 and S10). In 2011, there was no change in the incidence rate level of hypertension (change in rate =0.06 [95% CI, −0.16–0.27) but a leveling off of downward trend (change in yearly trend =0.09 [95% CI, 0.02–0.15). There was no change in the level or trend for incidence of major CVD events (change in rate =−0.001 [95% CI, −0.056–0.055]; change in yearly trend =0.010 [95% CI, −0.009–0.030]). Although these practices were selected for having high rates of out-of-office monitoring across the study period, a large increase in the use of out-of-office monitoring was also observed in this subgroup after 2011 (Figure S11).

## Discussion

The introduction of the NICE Hypertension guideline in England in 2011 was associated with a leveling out of downward trends in the incidence of diagnosed hypertension, although this may not be causal. In 2011, there was a step-change in the rate of ambulatory and home BP monitoring, in those with and without hypertension, suggesting changes in BP measurement occurred for diagnosis and monitoring. However, changes were modest in absolute terms implying perhaps issues with guideline implementation or coding of monitoring. By 2017, around a quarter of new diagnoses were accompanied by evidence of out-of-office monitoring. Contrary to previous predictions,^10^ there was little or no change in the incidence of cardiovascular morbidity or mortality.

### Strengths and Limitations

This was a large scale analysis of high-quality data representative of the UK population.^14^ The majority of excluded patients were those not registered at contributing practices or within the study age range during the study period (Figure 1). Other exclusions were those not eligible for data linkage (largely those registered at practices outside of England) and those without acceptable records for research (primarily those registered as temporary patients with unknown follow-up status). The age and sex profiles of those included/not on this basis were similar (data not shown),and since all people resident in the England are entitled to access national health services, we are confident that selection bias was limited. Our use of standardized rates further increases the applicability of the results to wider populations, even in the presence of differences in age and sex demographics.

We used diagnostic codes to define hypertension diagnoses which may have led to an underestimate of incidence.^20^ However, trends over time were similar when considering a broader definition, and patterns mirrored those observed in the United Kingdom for resistant hypertension.^21^ We could not use data from the Myocardial Infarction National Audit Project (a comprehensive register of patients experiencing MI) to ascertain fully validated MI outcomes. However, CPRD data is the most complete single source of information and when combined with hospital data as here, 92% of MI cases are identified.^22^ The effect of any under ascertainment of events in our composite outcomes is, therefore, likely to be limited. Reassuringly, we observed a decline in CVD-related mortality consistent with national statistics.^23^

Interrupted time series analysis cannot establish causality, as demonstrated by our finding that the incidence rate of asthma changed in 2011, which cannot plausibly be attributed to the hypertension guideline change. Our results may be influenced by coincidental factors including increases in general practitioner workload between 2007 and 2014^12^ without equivalent increases in staffing levels,^24^ which may have reduced access for patients, resulting in fewer diagnoses. Conversely, mortality rates declined and healthcare spending increased in the years before 2011, but both have since leveled off.^25^ Population-level improvements in health in the early years of our study may, therefore, not have been maintained. Nevertheless, our results were similar across analyses of CVD events and mortality regardless of the definition of CVD. Results for our primary outcome were also unaffected when adjusting for deprivation in addition to age and sex or in practices with greater use of out-of-office monitoring.

We chose to study major cardiovascular outcomes and mortality, to reflect the overall goal of BP lowering and powered the study to detect small changes in incidence levels and trends in the presence of autocorrelation. Previous studies in UK databases have been able to detect differences in CVD event and mortality rates in smaller numbers of patients with less follow-up.^26^ We are confident we could have detected changes in these outcomes in the medium term if they truly existed but cannot exclude the possibility that the effects of guideline change may only manifest in the longer term. Such lagged effects could be studied once further time has elapsed.

Guideline changes may have affected GP practices differently, depending on uptake. Models including random effects for GP practice were infeasible due to the large volumes of data in this study. This necessitated the use of yearly aggregate data in each age-gender strata and weighted analysis, but contemporary statistical software cannot implement random effects models with analytical weights. However, we were able to repeat analyses in practices with high levels of recorded out-of-office monitoring, and results in this subgroup were similar. Our use of age and sex as standardization variables meant we were unable to study patterns stratified by age or sex. Review evidence suggests that gender, in particular, is associated with the presence of masked and white coat hypertension,^27^ and therefore, the change in guidance may have had differential effects in men and women. Further work would be required to explore this.

Although we standardized our results for deprivation in sensitivity analyses, we did not adjust for other covariates that may influence the risk of hypertension, in particular body mass index. However, trends in body mass index over time are small (0.2 kg/m^2^ between 2007 and 2016),^28^ and results accounting for deprivation were similar to our main analyses, so further adjustment is unlikely to have altered our results.

### Comparisons With Existing Literature

Our finding that the incidence of hypertension has fallen since 2006 is consistent with observed downward trends in blood pressure.^29^ Considering the aging UK population and that the prevalence of hypertension increases with age, our finding is also consistent with studies showing that the overall prevalence of hypertension is stable.^30^ The decrease in the incidence of hypertension observed in this study may have been driven by an artificially inflated incidence rate shortly after the implementation of pay-for-performance indicators in 2004 in the United Kingdom. However, such an inflation was not observed when we repeated our analysis using data from 2003 onwards (not shown).

Although the change in guidance was associated with a change in the rate of out-of-office monitoring, observed changes were small in absolute terms. We also observed a decline in office BP measurement after 2011, which would be consistent with replacement of at least some of these measurements with out-of-office readings, as recommended. However, our findings suggest that the overwhelming majority of BP measurement is still recorded as performed in the clinic. This may be explained by a failure to implement guideline changes properly or from issues in coding. Previous research examining the impact of other guideline or policy changes have indeed shown limited impact on hypertension-related clinical practice.^16,31^ However, repeated UK surveys show that the vast majority of UK general practices now have access to home and ambulatory BP monitoring, with increases since 2011.^32^ Approximately 1 in 5 of all patients are also asked to monitor their BP at home.^33^ This suggests that the low rate of out-of-office monitoring in this study is more likely to reflect limited use of specific coding, rather than limited uptake of guidance. This appears to be a systematic issue as results were similar in practices where the use of out-of-office BP coding was high. This may be due to difficulty in using codes or the fact that BP-related pay-for-performance measures depend on clinic measurements.^34^

### Implications for Research and Practice

Our results indicate that concerns that the introduction of the NICE hypertension guideline in 2011 would result in major increases in avoidable cardiovascular events and deaths,^10^ appear unfounded, at least in the short-medium term. More recent guidelines in North America^35^ and Europe^36^ have taken up the UK NICE recommendations. We have found no evidence that such continued guidance would materially affect important patient outcomes. Further studies, in countries which have implemented similar guidance, or restricted to general practices which have fully adopted the guideline recommendations, are warranted to confirm our findings.

Our results indicate that the change in guidance was associated with a relative increase in hypertension diagnoses compared with that expected from pre-2011 trends, but further research would be required to understand the mechanism underpinning this. It is possible that the guideline change prompted closer monitoring of patients with borderline clinic BP readings who would have subsequently had hypertension ruled out (indicating white coat effects) or been diagnosed.

### Perspectives

For the diagnosis of hypertension, out-of-office blood pressure monitoring is more accurate^8^ and cost-effective^9^ compared with clinic BP monitoring. The results from this study suggest that the recommendation of out-of-office monitoring for hypertension diagnosis was not associated with observed negative consequences. By the end of the observation period, around a quarter of all new diagnoses were accompanied by recorded out-of-office measurement, but doubt remains regarding whether guidance was appropriately implemented or whether coding issues masked larger increases in the use of out-of-office BP measurement for the diagnosis of hypertension. The wider implementation of out-of-office monitoring, as has been recommended in the recent US, European and 2019 NICE hypertension guidelines, remains appropriate.

## Acknowledgments

The protocol was approved by the Independent Scientific Advisory Committee (ISAC) of the Medicines and Healthcare products Regulatory Agency (ISAC protocol number 17_239R; available from the authors on request). Ethics approval for observational research using the Clinical Practice Research Datalink with approval from ISAC was granted by a National Research Ethics Service committee (Trent Multi Research Ethics Committee, REC reference number 05/MRE04/87). All authors contributed to the study design, funding application, and ethical approval. S.L. Lay-Flurrie conducted data cleaning and analysis with input from R.J. Stevens and J.P. Sheppard. All authors interpreted results. S.L. Lay-Flurrie and R.J. McManus drafted the article which all authors then revised. S.L. Lay-Flurrie is the guarantor. The corresponding author attests that all listed authors meet authorship criteria and that no others meeting the criteria have been omitted.

## Sources of Funding

This study is funded by the National Institute for Health Research (NIHR) School for Primary Care Research (SPCR) (project reference 388). The views expressed are those of the author(s) and not necessarily those of the NIHR or the Department of Health and Social Care. J.P. Sheppard is funded by a Wellcome Trust/Royal Society Sir Henry Dale Fellowship (ref: 211182/Z/18/Z) and previously received support from the NIHR Oxford Collaborations for Leadership in Applied Research and Care (CLARHC), and the NIHR School for Primary Care Research. C. Mallen is funded by the NIHR Collaborations for Leadership in Applied Health Research and Care West Midlands and a NIHR Research Professorship in General Practice (NIHR-RP-2014-04-026). F.D.R. Hobbs acknowledges funding from the NIHR School for Primary Care Research, the NIHR CLARHC Oxford, the NIHR Oxford Biomedical Research Centre (BRC, University Hospitals Trust), and the NIHR Oxford Medtech and In-Vitro Diagnostics Co-operative (MIC). R.J. McManus received support from NIHR via Programme Grants for Applied Health research, the SPCR, the Oxford CLAHRC, and a Senior Investigator Award. J. Mant is an NIHR Senior Investigator. The study sponsors were not involved in any aspect of the study including study design, data collection, data analysis, and interpretation of data.

## Disclosures

All authors have completed the International Committee of Medical Journal Editors uniform disclosure form at www.icmje.org/coi_disclosure.pdf and declare: S.L. Lay-Flurrie reports grants from National Institute for Health Research, School for Primary Care Research, during the conduct of the study; R.J. McManus reports grants from National Institute for Health Research, during the conduct of the study and grants from Omron, outside the submitted work; and being a member of the 2019 National Institute for Health and Care Excellence Hypertension Guideline Committee; J.P. Sheppard reports grants from National Institute for Health Research and Wellcome Trust, during the conduct of the study; C. Heneghan reports grants from National Institute for Health Research and other expenses from World Health Organization, outside the submitted work, and is Editor in chief of the British Medical Journal Evidence-Based Medicine and an National Health Service Urgent Care general practitioner. The other authors report no conflicts.

## Supplementary Material

## Novelty and Significance

### What Is New?

- National guidelines in the United Kingdom recommended the use of out-of-office monitoring for hypertension diagnosis in 2011, but it was suggested this may increase rates of cardiovascular disease. This study is the first to formally evaluate the impact of these guideline changes on clinical outcomes at the population level.

### What is Relevant?

- Our study shows that changes to guidelines in 2011 were associated with a leveling off of downward trends in the incidence of hypertension and increases in the use of out-of-office monitoring. Changes were not associated with an increase in cardiovascular events, at least in the short-medium term.

### Summary

Based on current evidence, the continued recommendation of out-of-office monitoring for hypertension diagnosis is unlikely to affect hard patient outcomes.
