# Supplementary material for: Impact of Changes to National Hypertension Guidelines on Hypertension Management and Outcomes in the United Kingdom
Source: Hypertension. 2019 Dec 23;75(2):356–64. doi: 10.1161/HYPERTENSIONAHA.119.13926 (PMC7055938; doi:10.1161/HYPERTENSIONAHA.119.13926)

**ONLINE SUPPLEMENT**

Title: The impact of changes to national hypertension guidelines on hypertension management and outcomes in the UK

Authors:

Sarah L Lay-Flurrie, BSc MSc DPhil, University of Oxford

James P Sheppard, BSc PGCert PhD, University of Oxford

Richard J Stevens, BA MSc PhD, University of Oxford

Christian Mallen, BMedSci BMBS MMedSci MPhil PhD FRCGP FFPH, Keele University

Carl Heneghan, BM BCH MA MRCGP, DPhil, University of Oxford

FD Richard Hobbs, FRCP(Lon) FESC FRCP(Ed) FRCGP FMedSci, University of Oxford

Bryan Williams, MD FRCP FAHA FESC, University College London

Jonathan Mant, MA MSc MBBS MD FFPH FRCP(Ed) FMedSci, University of Cambridge

Richard J McManus, MA PhD MBBS FRCGP FRCP, University of Oxford

Short title: Impact of hypertension guideline change in the UK

Corresponding author: Sarah L Lay-Flurrie, 01865 289449, [sarah.lay-flurrie@phc.ox.ac.uk](mailto:sarah.lay-flurrie@phc.ox.ac.uk)

Nuffield Department of Primary Care Health Sciences, University of Oxford, Radcliffe Observatory Quarter, Woodstock Road, Oxford, OX2 6GG, United Kingdom.

**Extended methods**

*Study population*

Eligible patients entered the study on the earliest of the following dates: study start date (1st April 2006), date of 18th birthday, current registration date with practice and practice up-to-standard date. Patients exited the study on the earliest date of the following dates: study end date (31st March 2017), date of death, date of de-registration with the practice, date of last data download from practice and end of follow-up in linked ONS/ HES data.

*Sample size*

We intended to estimate both changes in the level of hypertension incidence and changes in trend. Research regarding sample size calculations for interrupted time series has shown that models including 60 data points, with equal pre- and post-intervention time periods, have more than 95% power to detect both changes in level and trend when autocorrelation is low (0.2 to 0.3), even when effect sizes are small.^1^ Based on our previous work examining the effect of policy changes on treatment for mild hypertension, where incidence rates were similar to those expected in this study,^2^ we expected autocorrelations to be approximately 0.25. As we have 120 data points in our time series (120 months over 10 years), we therefore expected adequate power (>80%) to detect even small changes in incidence level and trend.

*Outcomes*

The medical/ Read code list for diagnosis of hypertension (primary outcome) is given in Table S1. The specific definition of each secondary outcome is given below. Due to their size, code lists are not given here but are available from the authors on request.

New antihypertensive treatment prescription: defined as new treatment prescriptions in any eligible patients in a given month (numerator) divided by person-years of follow-up in the month in all eligible patients who have not previously been prescribed antihypertensive treatment (denominator). Antihypertensive medication was defined as: any of or combination of: calcium channel blocker, ACE inhibitor, Angiotensin Receptor Blocker, thiazide or thiazide like diuretic, beta blocker, alpha blocker, spironolactone, centrally acting antihypertensive or other antihypertensive not previously mentioned but listed in the current BNF.

Blood pressure monitoring (office, home and ambulatory): defined as all instances of blood pressure monitoring in any eligible patient in a given month (numerator) divided by person-years of follow-up in the month in all eligible patients (denominator).

Cardiovascular disease morbidity: defined as all new cases of cardiovascular disease in any eligible patients in a given month (numerator) divided by person-years of follow-up in the month in all eligible patients who have not previously had a case of cardiovascular disease (denominator). Cardiovascular events were determined from the first clinical code recorded in the primary care record or linked hospital or mortality record. Major cardiovascular events were defined “hard” cardiovascular events comprising any of cardiovascular death, myocardial infarction, or stroke. Sensitivity analyses examined the impact of including unstable angina, coronary artery bypass graft and heart failure in the definition.

Cardiovascular mortality: defined as all cardiovascular deaths in any eligible patients in a given month (numerator) divided by person-years of follow-up in the month in all eligible patients (still alive) (denominator). Date and cause of death were determined from linked mortality data.

All-cause mortality: defined as all deaths in any eligible patients in a given month (numerator) divided by person-years of follow-up in the month in all eligible patients (still alive) (denominator). Date of death was determined from linked mortality data.

*Data cleaning and analysis*

SLF had access to the full CPRD database and conducted all data cleaning and analysis. Linked data for eligible patients only was provided directly by CPRD. Code lists and programming code is available from the authors on request.

*Data cleaning*

All data was drawn from the coded primary care data, with the following exceptions. Date and cause of death was drawn from linked mortality data only. Cardiovascular outcomes were defined according to the earliest record of a cardiovascular event in the primary care record, linked inpatient hospital data or any cardiovascular cause in linked mortality data. Ethnicity data was derived from primary care records and from hospital episode statistics data where missing in the primary care record. The most recent record of ethnic group was used. Deprivation data was drawn from linked index of multiple deprivation data only.

In all analyses, blood pressure readings not explicitly defined as readings taken at home or through ambulatory BP monitoring were classified as clinic measurements. In analyses of the rate of BP monitoring, repeat clinic measurements on the same day were counted as a single measurement. Repeat home/ ambulatory measurements within a week were also only counted as a single instance of home/ ambulatory monitoring, allowing for the fitting and return of devices. For the sensitivity analysis defining hypertension based on blood pressure readings, BP readings below 70/30 mm Hg and above 260/140 mm Hg were deemed implausible and removed. For multiple measurements on the same day, the lowest BP measurement was used.

**References**

1. Zhang F, Wagner AK, Ross-Degnan D. Simulation-based power calculation for designing interrupted time series analyses of health policy interventions. *J Clin Epidemiol*. 2011;64(11):1252-1261. doi:10.1016/J.JCLINEPI.2011.02.007

2. Sheppard JP, Stevens S, Stevens RJ, et al. Association of guideline and policy changes with incidence of lifestyle advice and treatment for uncomplicated mild hypertension in primary care: a longitudinal cohort study in the Clinical Practice Research Datalink. *BMJ Open*. 2018;8(9):e021827. doi:10.1136/bmjopen-2018-021827

Table S1: Hypertension diagnostic codes used to define primary outcome

| **Medical code** | **Read code** | **Read Term** |
| --- | --- | --- |
| 16565 | 6627 | Good hypertension control |
| 27511 | 6628 | Poor hypertension control |
| 30776 | 6629 | Hypertension:follow-up default |
| 32976 | 6146200 | Hypertension induced by oral contraceptive pill |
| 2666 | 14A2.00 | H/O: hypertension |
| 109611 | 661M600 | Hypertension self-management plan agreed |
| 110631 | 661N600 | Hypertension self-management plan review |
| 4444 | 662..12 | Hypertension monitoring |
| 18590 | 662b.00 | Moderate hypertension control |
| 18482 | 662c.00 | Hypertension six month review |
| 19070 | 662d.00 | Hypertension annual review |
| 21826 | 662F.00 | Hypertension treatm. started |
| 13188 | 662G.00 | Hypertensive treatm.changed |
| 12948 | 662H.00 | Hypertension treatm.stopped |
| 3425 | 662O.00 | On treatment for hypertension |
| 13186 | 662P.00 | Hypertension monitoring |
| 102406 | 662P000 | Hypertension 9 month review |
| 109771 | 662P100 | Telehealth hypertension monitoring |
| 71433 | 66b2.00 | Hypertension monitoring not required |
| 204 | G2...00 | Hypertensive disease |
| 8732 | G2...11 | BP - hypertensive disease |
| 799 | G20..00 | Essential hypertension |
| 351 | G20..11 | High blood pressure |
| 107704 | G20..12 | Primary hypertension |
| 15377 | G200.00 | Malignant essential hypertension |
| 1894 | G201.00 | Benign essential hypertension |
| 4372 | G202.00 | Systolic hypertension |
| 83473 | G203.00 | Diastolic hypertension |
| 10818 | G20z.00 | Essential hypertension NOS |
| 3712 | G20z.11 | Hypertension NOS |
| 7329 | G24..00 | Secondary hypertension |
| 31755 | G240.00 | Secondary malignant hypertension |
| 59383 | G240000 | Secondary benign renovascular hypertension |
| 73293 | G240z00 | Secondary malignant hypertension NOS |
| 57288 | G241.00 | Secondary benign hypertension |
| 25371 | G241000 | Secondary benign renovascular hypertension |
| 51635 | G241z00 | Secondary benign hypertension NOS |
| 34744 | G244.00 | Hypertension secondary to endocrine disorders |
| 16059 | G24z.00 | Secondary hypertension NOS |
| 31387 | G24z000 | Secondary renovascular hypertension NOS |
| 31341 | G24z100 | Hypertension secondary to drug |
| 42229 | G24zz00 | Secondary hypertension NOS |
| 105371 | G25..00 | Stage 1 hypertension (NICE - Nat Ins for Hth Clin Excl 2011) |
| 105316 | G25..11 | Stage 1 hypertension |
| 108136 | G250.00 | Stage 1 hyperten (NICE 2011) without evidnce end organ damge |
| 109797 | G251.00 | Stage 1 hyperten (NICE 2011) with evidnce end organ damge |
| 105989 | G26..00 | Severe hypertension (Nat Inst for Health Clinical Ex 2011) |
| 105487 | G26..11 | Severe hypertension |
| 105480 | G27..00 | Hypertension resistant to drug therapy |
| 105274 | G28..00 | Stage 2 hypertension (NICE - Nat Ins for Hth Clin Excl 2011) |
| 18765 | G2y..00 | Other specified hypertensive disease |
| 7057 | G2z..00 | Hypertensive disease NOS |
| 69753 | Gyu2.00 | [X]Hypertensive diseases |
| 102458 | Gyu2000 | [X]Other secondary hypertension |
| 97533 | Gyu2100 | [X]Hypertension secondary to other renal disorders |

Table S2: Crude incidence rates of hypertension (per 100 person years (%)) in each age-sex strata by year:

| **Gender** | **Age group** | **2006/7** | **2007/8** | **2008/9** | **2009/10** | **2010/11** | **2011/12** | **2012/13** | **2013/14** | **2014/15** | **2015/16** | **2016/17** |
| --- | --- | --- | --- | --- | --- | --- | --- | --- | --- | --- | --- | --- |
| **Men** | **18-24** | 0.07 | 0.05 | 0.04 | 0.04 | 0.04 | 0.04 | 0.04 | 0.04 | 0.05 | 0.05 | 0.05 |
| **Men** | **25-44** | 0.51 | 0.49 | 0.51 | 0.46 | 0.43 | 0.41 | 0.43 | 0.46 | 0.38 | 0.41 | 0.41 |
| **Men** | **45-54** | 1.93 | 1.85 | 1.81 | 1.64 | 1.57 | 1.55 | 1.57 | 1.54 | 1.45 | 1.48 | 1.57 |
| **Men** | **55-64** | 3.50 | 3.26 | 3.28 | 2.97 | 2.80 | 2.62 | 2.73 | 2.63 | 2.38 | 2.33 | 2.51 |
| **Men** | **65-74** | 5.06 | 4.44 | 4.34 | 4.11 | 3.85 | 3.47 | 3.55 | 3.41 | 2.97 | 3.10 | 3.15 |
| **Men** | **75-84** | 5.07 | 4.45 | 4.21 | 3.86 | 3.69 | 3.08 | 3.35 | 3.25 | 3.14 | 3.09 | 3.30 |
| **Men** | **85+** | 3.39 | 3.15 | 3.10 | 2.70 | 2.55 | 2.32 | 2.61 | 2.53 | 2.40 | 2.45 | 2.33 |
| **Women** | **18-24** | 0.08 | 0.07 | 0.08 | 0.06 | 0.07 | 0.07 | 0.06 | 0.05 | 0.07 | 0.08 | 0.06 |
| **Women** | **25-44** | 0.43 | 0.41 | 0.44 | 0.40 | 0.36 | 0.34 | 0.37 | 0.37 | 0.36 | 0.34 | 0.34 |
| **Women** | **45-54** | 1.60 | 1.54 | 1.46 | 1.36 | 1.22 | 1.14 | 1.22 | 1.24 | 1.11 | 1.24 | 1.32 |
| **Women** | **55-64** | 2.83 | 2.57 | 2.56 | 2.27 | 2.12 | 2.02 | 1.94 | 1.95 | 1.75 | 1.84 | 1.92 |
| **Women** | **65-74** | 5.11 | 4.57 | 4.17 | 3.72 | 3.45 | 3.14 | 3.06 | 3.20 | 2.74 | 2.69 | 2.81 |
| **Women** | **75-84** | 6.33 | 5.91 | 5.58 | 4.61 | 4.52 | 4.11 | 3.88 | 4.04 | 3.71 | 4.03 | 3.86 |
| **Women** | **85+** | 4.36 | 3.77 | 3.96 | 3.30 | 3.12 | 3.02 | 3.17 | 3.12 | 2.61 | 3.03 | 3.49 |

Table S3: Results from interrupted time series analysis of age and sex standardized rates of treated and untreated incident hypertension between April 2006 and March 2017, with interruption between April 2011 and March 2012

|  | **Treated hypertension** | | | **Untreated hypertension** | | |
| --- | --- | --- | --- | --- | --- | --- |
| **Model parameter** | **Estimate (per 100 person-years)** | **95% confidence interval** | | **Estimate (per 100 person-years)** | **95% confidence interval** | |
| **Initial rate (April 2006)** | 1.666 | 1.556 | 1.776 | 0.438 | 0.395 | 0.480 |
| **Initial trend per year (April 2006 - March 2011)** | -0.081 | -0.117 | -0.045 | -0.027 | -0.039 | -0.015 |
| **Predicted rate (April 2012)** | 1.174 | 1.042 | 1.306 | 0.275 | 0.238 | 0.312 |
| **Post-intervention rate (April 2012)** | 1.141 | 1.061 | 1.221 | 0.318 | 0.297 | 0.339 |
| **Post-intervention trend per year (April 2012 - March 2017)** | -0.018 | -0.048 | 0.012 | 0.003 | -0.004 | 0.011 |
| **Change in rate** | -0.033 | -0.187 | 0.121 | 0.043 | 0.000 | 0.086 |
| **Change in trend** | 0.063 | 0.016 | 0.110 | 0.030 | 0.016 | 0.044 |
| **End rate (March 2017)** | 1.053 | 0.965 | 1.142 | 0.334 | 0.310 | 0.359 |

Table S4: Results from interrupted time series analysis of age and sex standardized rates incident hypertension between April 2006 and March 2017, with interruption between April 2011 and March 2012 (sensitivity analysis with more sensitive definition of hypertension)

| **Model parameter** | **Estimate (per 100 person-years)** | **95% confidence interval** | |
| --- | --- | --- | --- |
| **Initial rate (April 2006)** | 3.205 | 3.014 | 3.396 |
| **Initial trend per year (April 2006 - March 2011)** | -0.124 | -0.187 | -0.061 |
| **Predicted rate (April 2012)** | 2.452 | 2.223 | 2.681 |
| **Post-intervention rate (April 2012)** | 2.433 | 2.289 | 2.577 |
| **Post-intervention trend per year (April 2012 - March 2017)** | -0.027 | -0.077 | 0.024 |
| **Change in rate** | -0.019 | -0.289 | 0.252 |
| **Change in trend** | 0.097 | 0.016 | 0.178 |
| **End rate (March 2017)** | 2.301 | 2.157 | 2.444 |

**Table S5**: Results from interrupted time series analysis of age and sex standardized rates of blood pressure monitoring between April 2006 and March 2017, with interruption between April 2011 and March 2012

|  | **Home** | | | **ABPM** | | | **Office** | | |
| --- | --- | --- | --- | --- | --- | --- | --- | --- | --- |
| **Model parameter** | **Estimate** | **95% confidence interval** | | **Estimate** | **95% confidence interval** | | **Estimate** | **95% confidence interval** | |
| **Initial rate (April 2006)** | 0.041 | 0.032 | 0.050 | 0.308 | 0.282 | 0.334 | 82.789 | 78.888 | 86.689 |
| **Initial trend per year**  **(April 2006 - March 2011)** | 0.017 | 0.013 | 0.020 | 0.014 | 0.004 | 0.024 | 1.111 | -0.269 | 2.491 |
| **Predicted rate**  **(April 2012)** | 0.142 | 0.129 | 0.154 | 0.393 | 0.355 | 0.431 | 89.548 | 84.178 | 94.918 |
| **Post-intervention rate**  **(April 2012)** | 0.278 | 0.237 | 0.318 | 0.908 | 0.835 | 0.982 | 86.420 | 82.589 | 90.251 |
| **Post-intervention trend per year**  **(April 2012 - March 2017)** | 0.101 | 0.084 | 0.119 | -0.013 | -0.037 | 0.011 | -2.785 | -4.069 | -1.501 |
| **Change in rate** | 0.136 | 0.093 | 0.178 | 0.515 | 0.433 | 0.598 | -3.128 | -9.724 | 3.469 |
| **Change in trend** | 0.085 | 0.067 | 0.102 | -0.027 | -0.053 | -0.001 | -3.896 | -5.781 | -2.011 |
| **End rate**  **(March 2017)** | 0.775 | 0.717 | 0.834 | 0.847 | 0.787 | 0.907 | 72.727 | 69.367 | 76.088 |

**Table S6: Results from interrupted time series analysis of age and sex standardized rates of incident cardiovascular disease, cardiovascular mortality and all-cause mortality (per 100 person-years)** between April 2006 and March 2017, with interruption between April 2011 and March 2012

|  | **Major CVD events** | | | **Any CVD event** | | | **Major CVD mortality** | | | **Any CVD mortality** | | | **All-cause mortality** | | |
| --- | --- | --- | --- | --- | --- | --- | --- | --- | --- | --- | --- | --- | --- | --- | --- |
| **Model parameter** | **Estimate** | **95% confidence interval** | | **Estimate** | **95% confidence interval** | | **Estimate** | **95% confidence interval** | | **Estimate** | **95% confidence interval** | | **Estimate** | **95% confidence interval** | |
| **Initial rate (April 2006)** | 0.539 | 0.519 | 0.559 | 1.316 | 1.274 | 1.358 | 0.155 | 0.145 | 0.166 | 0.325 | 0.305 | 0.345 | 1.316 | 1.241 | 1.391 |
| **Initial trend per year**  **(April 2006 - March 2011)** | 0.001 | -0.005 | 0.008 | 0.016 | 0.002 | 0.030 | -0.009 | -0.012 | -0.006 | -0.017 | -0.023 | -0.011 | -0.032 | -0.057 | -0.007 |
| **Predicted rate**  **(April 2012)** | 0.546 | 0.522 | 0.571 | 1.413 | 1.360 | 1.466 | 0.101 | 0.089 | 0.114 | 0.220 | 0.196 | 0.244 | 1.122 | 1.025 | 1.219 |
| **Post-intervention rate**  **(April 2012)** | 0.530 | 0.508 | 0.551 | 1.369 | 1.321 | 1.417 | 0.101 | 0.093 | 0.108 | 0.204 | 0.191 | 0.217 | 1.109 | 1.043 | 1.176 |
| **Post-intervention trend per year (April 2012 - March 2017)** | -0.003 | -0.011 | 0.005 | -0.001 | -0.019 | 0.017 | -0.004 | -0.006 | -0.001 | -0.009 | -0.014 | -0.004 | -0.015 | -0.040 | 0.010 |
| **Change in rate** | -0.017 | -0.049 | 0.016 | -0.044 | -0.115 | 0.027 | -0.001 | -0.015 | 0.014 | -0.017 | -0.044 | 0.011 | -0.013 | -0.130 | 0.105 |
| **Change in trend** | -0.004 | -0.014 | 0.006 | -0.017 | -0.039 | 0.006 | 0.005 | 0.001 | 0.010 | 0.008 | 0.000 | 0.016 | 0.017 | -0.019 | 0.052 |
| **End rate**  **(March 2017)** | 0.515 | 0.492 | 0.537 | 1.364 | 1.313 | 1.415 | 0.083 | 0.075 | 0.092 | 0.160 | 0.145 | 0.175 | 1.035 | 0.956 | 1.113 |

Table S7: Results from interrupted time series analysis of age and sex standardized rates of new and overall antihypertensive medication use between April 2006 and March 2017, with interruption between April 2011 and March 2012

|  | **New use** | | |
| --- | --- | --- | --- |
| **Model parameter** | **Estimate (per 100 person-years)** | **95% confidence interval** | |
| **Initial rate (April 2006)** | 3.746 | 3.554 | 3.937 |
| **Initial trend per year (April 2006 - March 2011)** | -0.068 | -0.135 | 0.000 |
| **Predicted rate (April 2012)** | 3.335 | 3.080 | 3.589 |
| **Post-intervention rate (April 2012)** | 3.229 | 3.069 | 3.390 |
| **Post-intervention trend per year (April 2012 - March 2017)** | 0.028 | -0.030 | 0.087 |
| **Change in rate** | -0.105 | -0.406 | 0.196 |
| **Change in trend** | 0.096 | 0.007 | 0.185 |
| **End rate (March 2017)** | 3.369 | 3.198 | 3.539 |

Table S8: Results from interrupted time series analysis of age and sex standardized rates for outcomes in patients with and without hypertension, between April 2007 and March 2017

|  | **In patients with hypertension** | | | **In patients without hypertension** | | |
| --- | --- | --- | --- | --- | --- | --- |
| **Model parameter** | **Estimate (per 100 person-years)** | **95% confidence interval** | | **Estimate (per 100 person-years)** | **95% confidence interval** | |
| **Ambulatory BP monitoring** | | | | | | |
| Initial rate (April 2007) | 3.888 | 3.353 | 4.422 | 0.272 | 0.251 | 0.293 |
| Initial trend per year (April 2007 - March 2011) | -0.171 | -0.241 | -0.100 | 0.001 | -0.002 | 0.004 |
| Predicted rate (April 2012) | 1.285 | 0.665 | 1.905 | 0.288 | 0.254 | 0.323 |
| Post-intervention rate (April 2012) | 3.486 | 3.099 | 3.873 | 0.787 | 0.719 | 0.856 |
| Post-intervention trend per year (April 2012 - March 2017) | -0.027 | -0.071 | 0.017 | -0.002 | -0.009 | 0.006 |
| Change in rate | 2.201 | 1.470 | 2.932 | 0.499 | 0.422 | 0.575 |
| Change in trend | 0.144 | 0.061 | 0.227 | -0.003 | -0.011 | 0.005 |
| End rate (March 2017) | 3.092 | 2.696 | 3.487 | 0.761 | 0.704 | 0.818 |
| **Home BP monitoring** | | | | | | |
| Initial rate (April 2007) | 0.815 | 0.645 | 0.985 | 0.051 | 0.045 | 0.057 |
| Initial trend per year (April 2007 - March 2011) | -0.010 | -0.034 | 0.013 | 0.001 | 0.000 | 0.002 |
| Predicted rate (April 2012) | 0.658 | 0.436 | 0.880 | 0.070 | 0.062 | 0.077 |
| Post-intervention rate (April 2012) | 1.209 | 0.989 | 1.428 | 0.196 | 0.165 | 0.227 |
| Post-intervention trend per year (April 2012 - March 2017) | 0.130 | 0.101 | 0.159 | 0.016 | 0.012 | 0.021 |
| Change in rate | 0.551 | 0.239 | 0.863 | 0.126 | 0.095 | 0.158 |
| Change in trend | 0.140 | 0.103 | 0.177 | 0.015 | 0.011 | 0.020 |
| End rate (March 2017) | 3.128 | 2.830 | 3.425 | 0.439 | 0.397 | 0.481 |
| **Office BP monitoring** | | | | | | |
| Initial rate (April 2007) | 349.590 | 332.804 | 366.376 | 78.221 | 74.464 | 81.978 |
| Initial trend per year (April 2007 - March 2011) | -7.979 | -10.324 | -5.634 | -0.283 | -0.835 | 0.269 |
| Predicted rate (April 2012) | 227.906 | 205.802 | 250.011 | 73.903 | 68.373 | 79.433 |
| Post-intervention rate (April 2012) | 242.229 | 231.905 | 252.553 | 74.099 | 70.882 | 77.316 |
| Post-intervention trend per year (April 2012 - March 2017) | -4.958 | -6.121 | -3.795 | -0.813 | -1.177 | -0.448 |
| Change in rate | 14.322 | -10.074 | 38.719 | 0.195 | -6.202 | 6.593 |
| Change in trend | 3.021 | 0.403 | 5.639 | -0.530 | -1.191 | 0.132 |
| End rate (March 2017) | 169.096 | 159.724 | 178.469 | 62.110 | 59.206 | 65.015 |
| **Hard CVD events** | | | | | | |
| Initial rate (April 2007) | 0.770 | 0.690 | 0.850 | 0.514 | 0.494 | 0.535 |
| Initial trend per year (April 2007 - March 2011) | 0.002 | -0.008 | 0.012 | 0.002 | -0.001 | 0.004 |
| Predicted rate (April 2012) | 0.801 | 0.719 | 0.884 | 0.540 | 0.513 | 0.568 |
| Post-intervention rate (April 2012) | 0.719 | 0.649 | 0.789 | 0.515 | 0.494 | 0.537 |
| Post-intervention trend per year (April 2012 - March 2017) | 0.000 | -0.008 | 0.008 | -0.002 | -0.004 | 0.001 |
| Change in rate | -0.082 | -0.191 | 0.026 | -0.025 | -0.060 | 0.010 |
| Change in trend | -0.002 | -0.015 | 0.010 | -0.003 | -0.007 | 0.001 |
| End rate (March 2017) | 0.714 | 0.653 | 0.775 | 0.493 | 0.469 | 0.516 |
| **First antihypertensive use** | | | | | | |
| Initial rate (April 2007) | 46.445 | 43.337 | 49.553 | 3.429 | 3.278 | 3.580 |
| Initial trend per year (April 2007 - March 2011) | -2.393 | -2.818 | -1.967 | -0.014 | -0.039 | 0.011 |
| Predicted rate (April 2012) | 9.957 | 6.104 | 13.811 | 3.218 | 2.949 | 3.486 |
| Post-intervention rate (April 2012) | 19.796 | 18.292 | 21.300 | 3.067 | 2.915 | 3.220 |
| Post-intervention trend per year (April 2012 - March 2017) | -0.366 | -0.545 | -0.186 | 0.010 | -0.008 | 0.029 |
| Change in rate | 9.839 | 5.702 | 13.975 | -0.150 | -0.459 | 0.158 |
| Change in trend | 2.027 | 1.565 | 2.489 | 0.024 | -0.007 | 0.055 |
| End rate (March 2017) | 14.405 | 12.819 | 15.991 | 3.220 | 3.057 | 3.383 |

**Figure S1: Age and sex standardized incidence of asthma (per 100 person-years) between April 2007 and March 2017 with interruption between April 2011 and March 2012.**
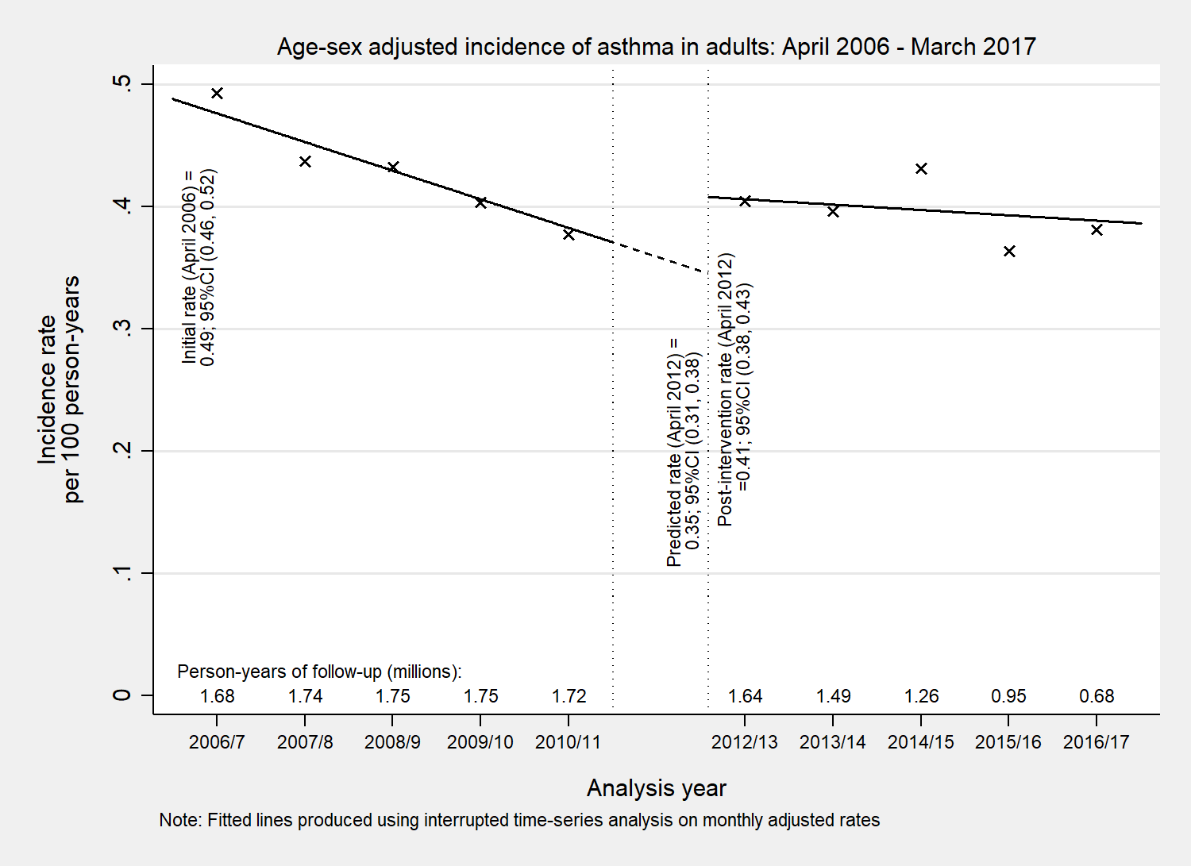


Figure S2: Age and sex standardized rate of new antihypertensive medication use (per 100 person-years) between April 2006 and March 2017 with interruption between April 2011 and March 2012.


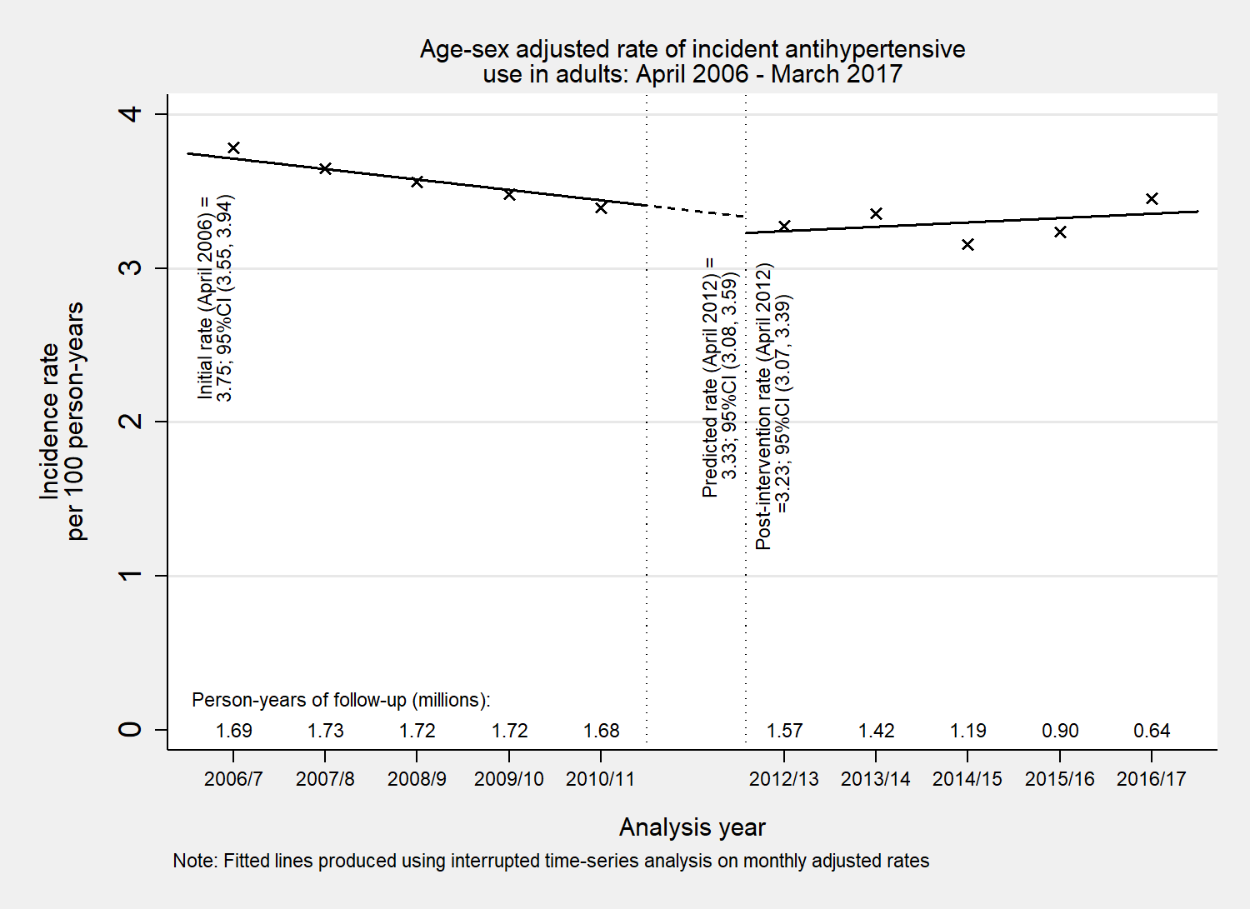


**Figure S3: Age and sex standardized rate of use of at least one antihypertensive medication by class (per 100 person-years) between April 2006 and March 2017 with interruption between April 2011 and March 2012.**


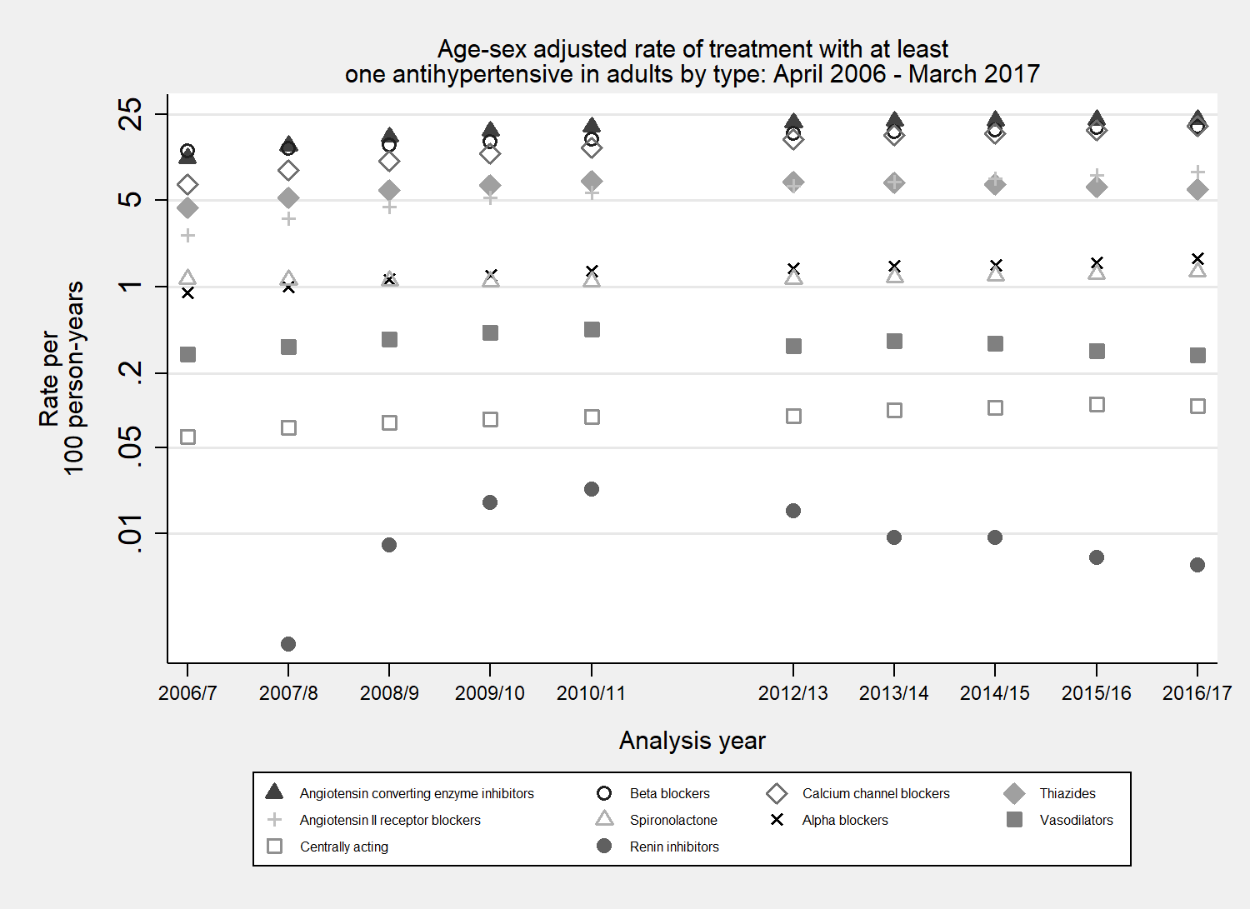


**Figure S4: Age and sex standardized rate of ambulatory BP monitoring (per 100 person-years), in hypertensive and normotensive patients, between April 2007 and March 2017 with interruption between April 2011 and March 2012.**


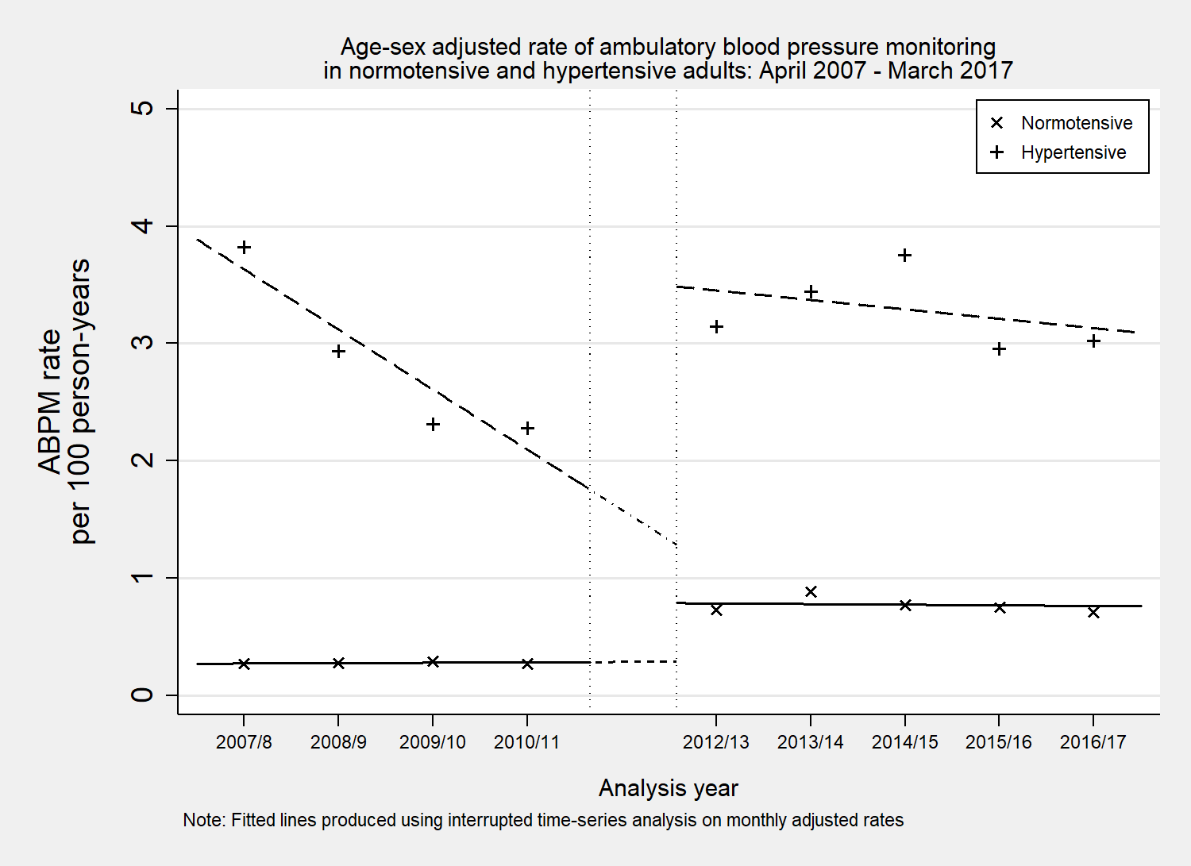


**Figure S5: Age and sex standardized rate of home BP monitoring (per 100 person-years), in hypertensive and normotensive patients, between April 2007 and March 2017 with interruption between April 2011 and March 2012.**


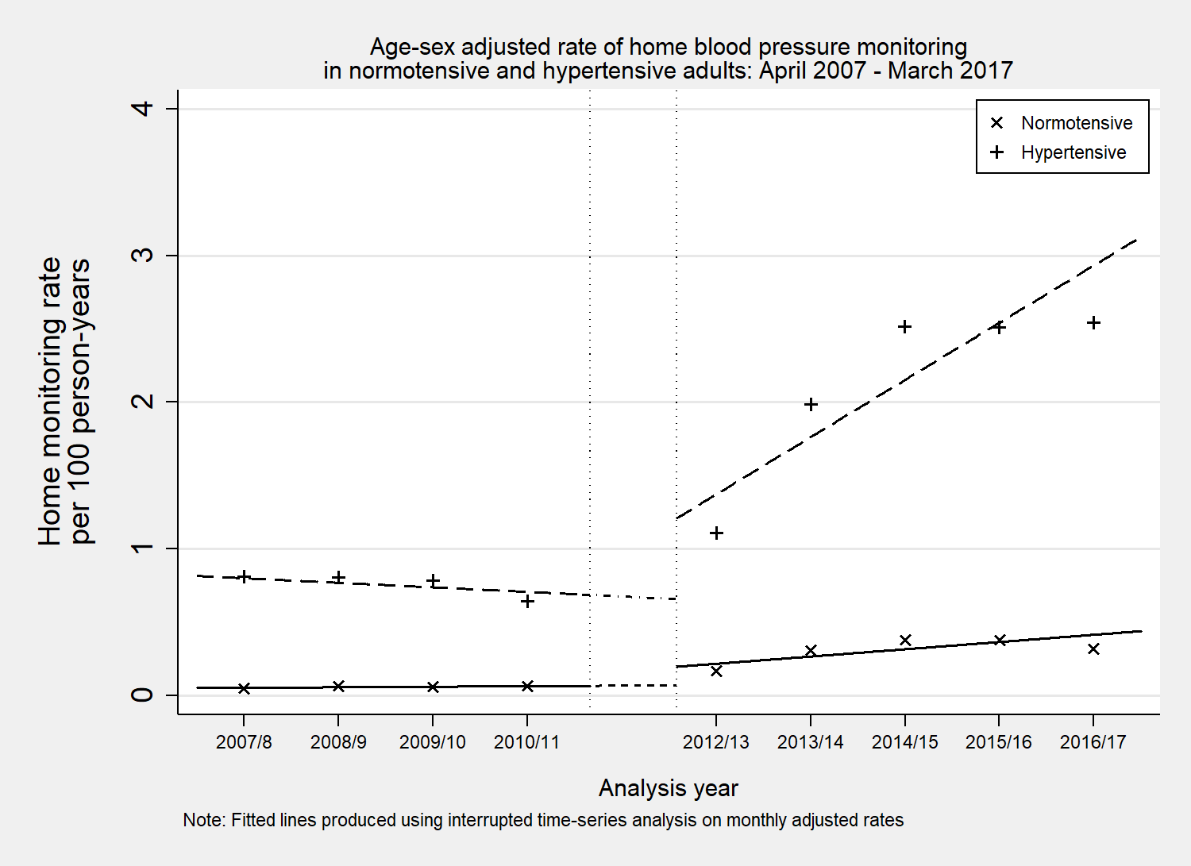


**Figure S6: Age and sex standardized rate of office BP monitoring (per 100 person-years), in hypertensive and normotensive patients, between April 2007 and March 2017 with interruption between April 2011 and March 2012.**


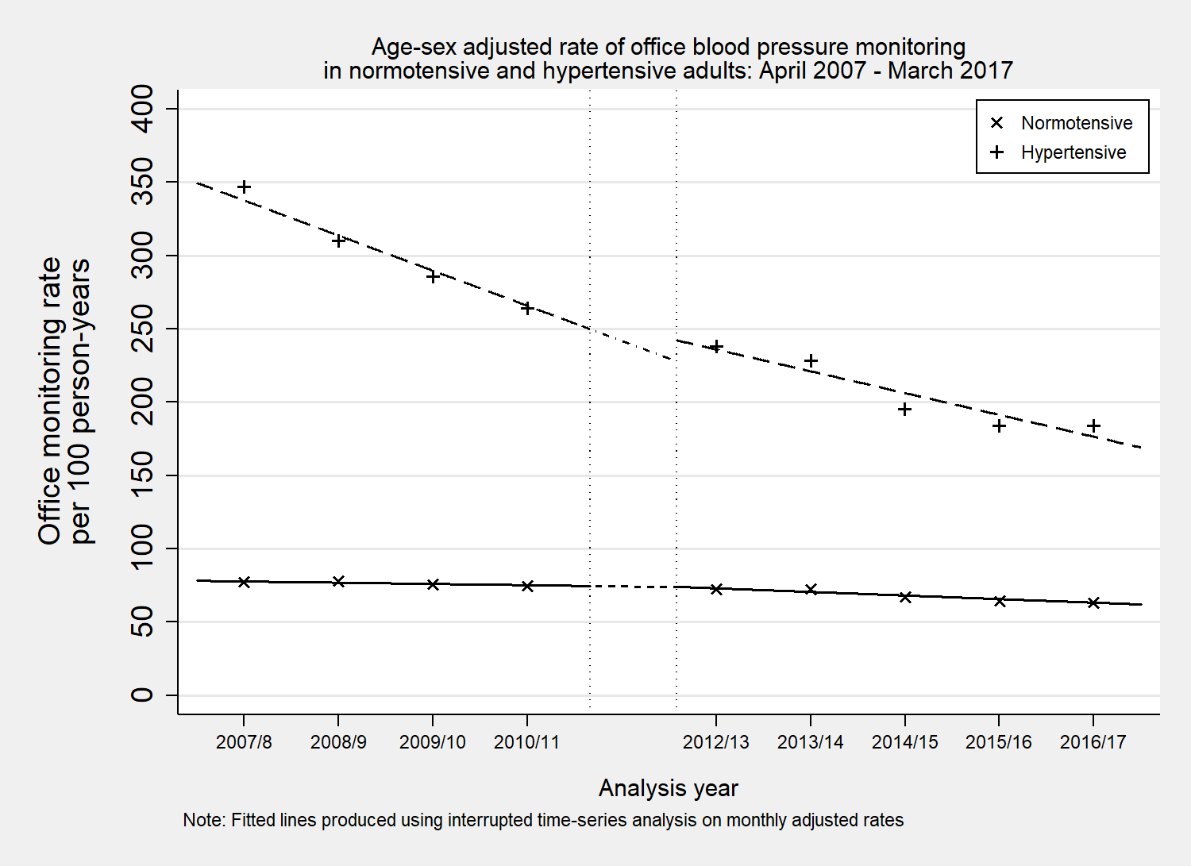


**Figure S7: Age and sex standardized rate of major CVD events (per 100 person-years), in hypertensive and normotensive patients, between April 2007 and March 2017 with interruption between April 2011 and March 2012.**


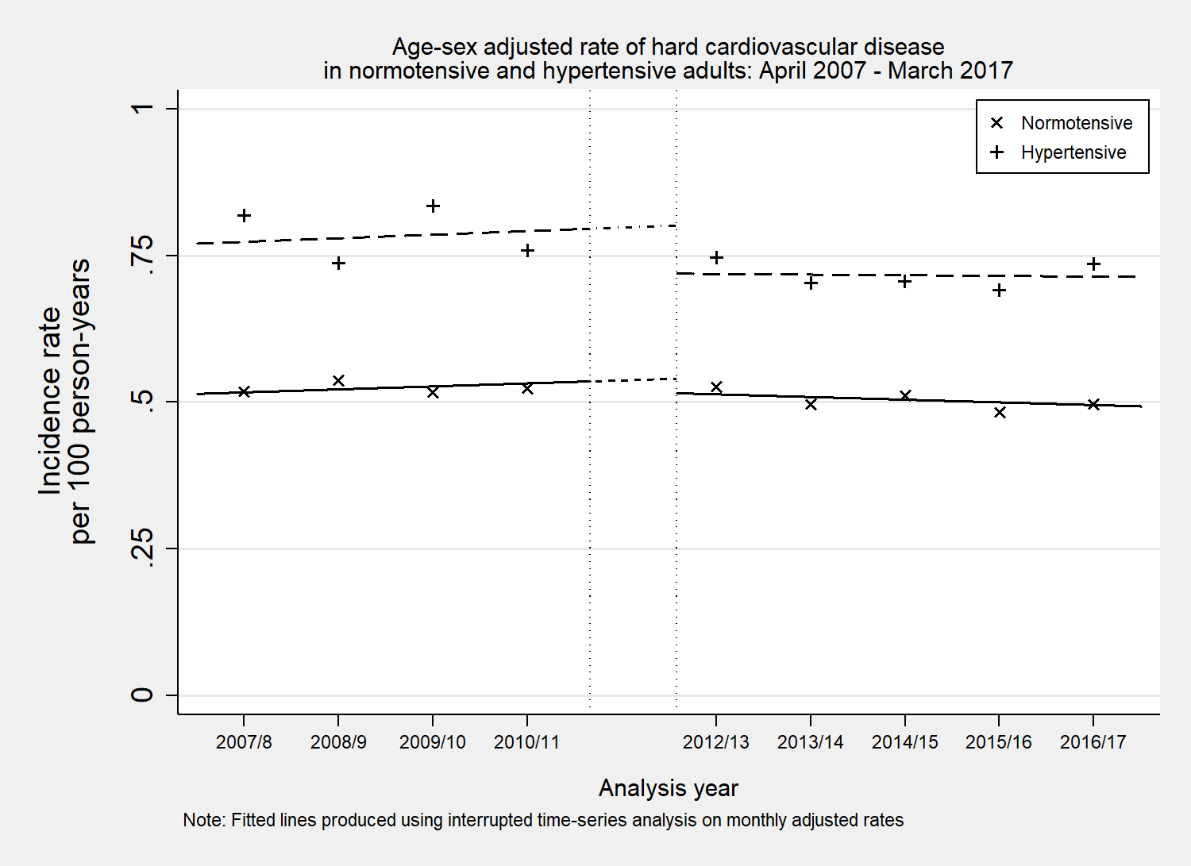


**Figure S8: Age and sex standardized rate of new antihypertensive use (per 100 person-years), in hypertensive and normotensive patients, between April 2007 and March 2017 with interruption between April 2011 and March 2012.**


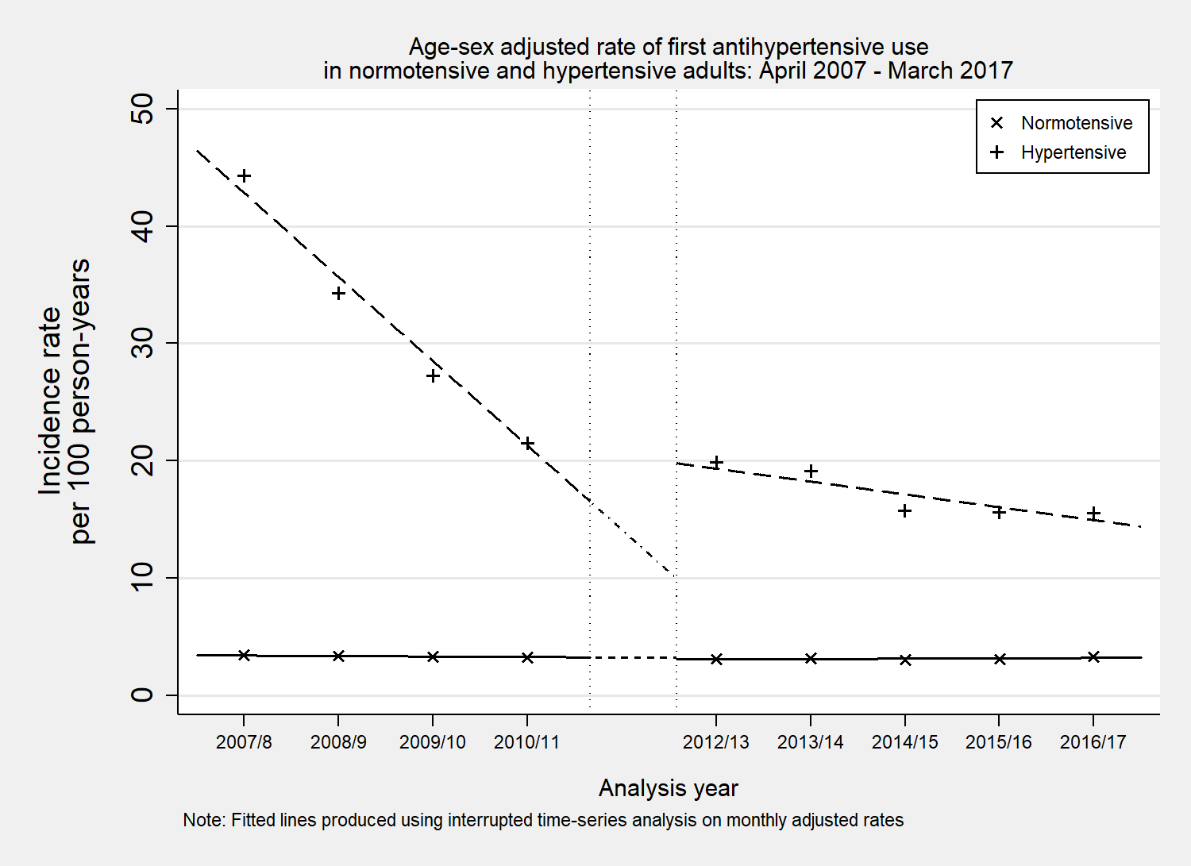


**Figure S9: Age and sex standardized incidence of hypertension (per 100 person-years) between April 2007 and March 2017 with interruption between April 2011 and March 2012 (subset of practices in top 20% of out-of-office monitoring rate).**


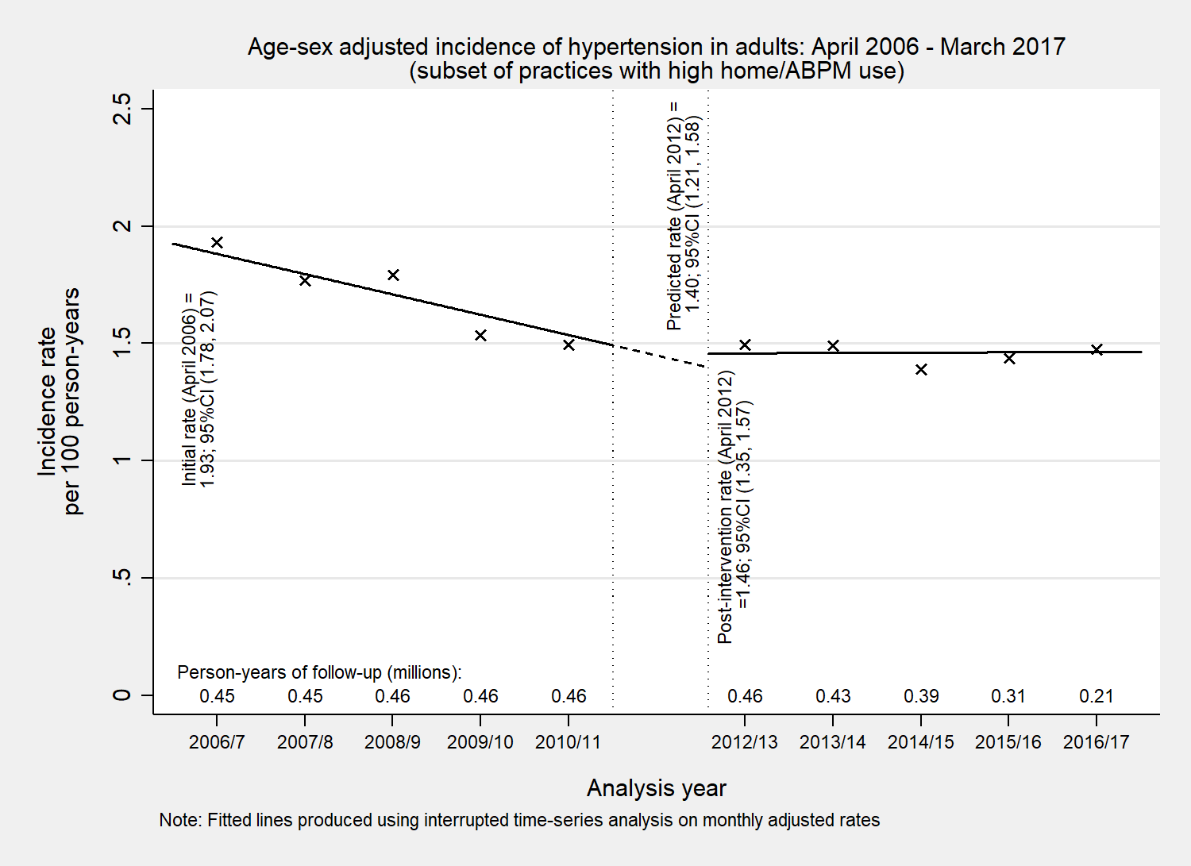


**Figure S10: Age and sex standardized incidence of major CVD events (per 100 person-years) between April 2007 and March 2017 with interruption between April 2011 and March 2012 (subset of practices in top 20% of out-of-office monitoring rate).**

**
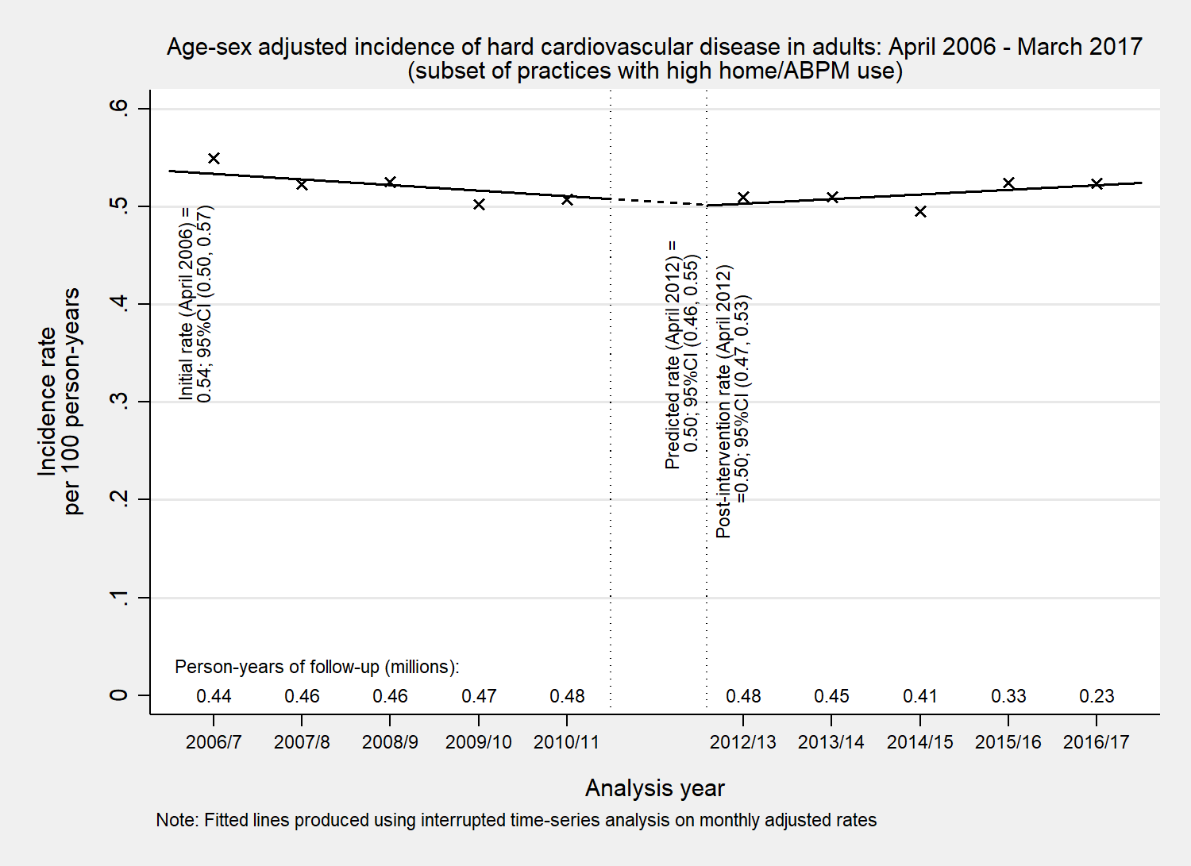
**

**Figure S11: Age and sex standardized rate of out-of-office monitoring events (per 100 person-years) between April 2007 and March 2017 with interruption between April 2011 and March 2012 (subset of practices in top 20% of out-of-office monitoring rate).**


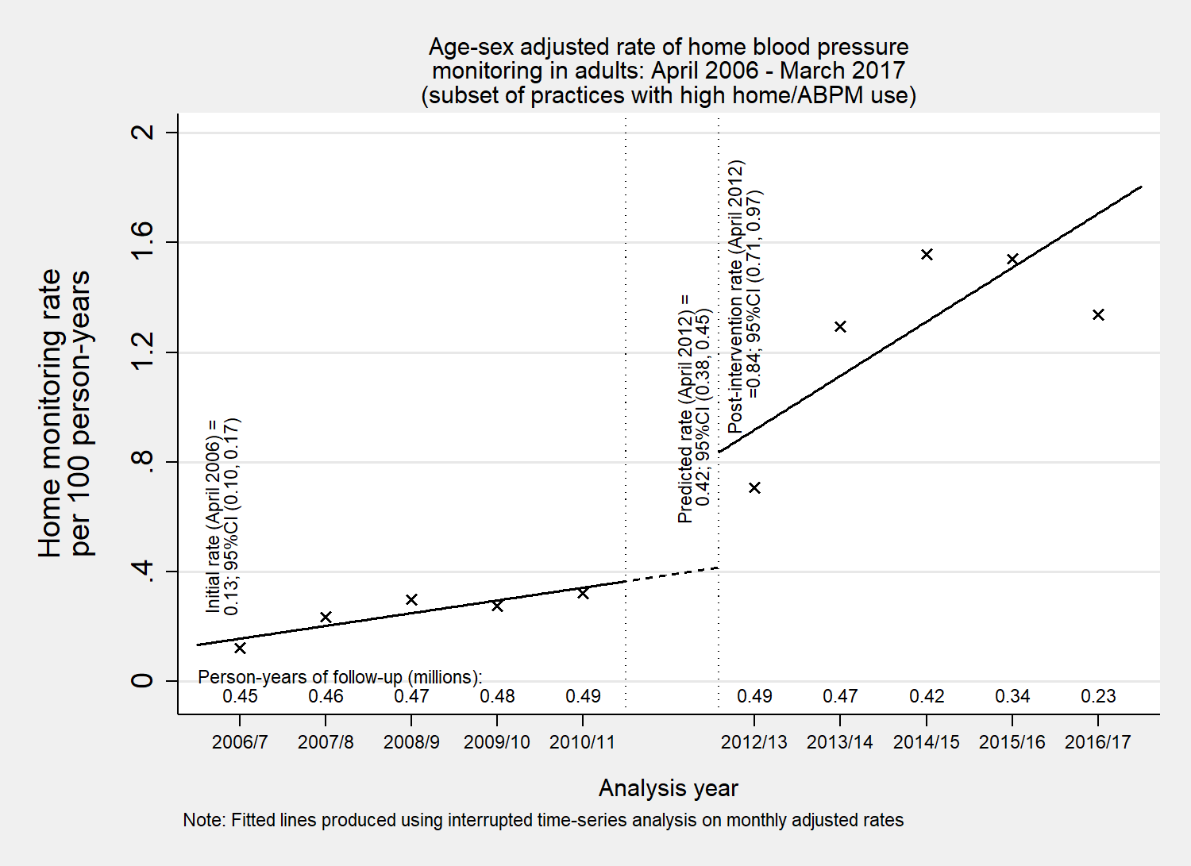

Supplement: Supplementary file 1 [file hyp-75-356-s001.doc]
