# Supplementary figures and images for: Impact of Changes to National Hypertension Guidelines on Hypertension Management and Outcomes in the United Kingdom
Source: Hypertension. 2019 Dec 23;75(2):356–64. doi: 10.1161/HYPERTENSIONAHA.119.13926 (PMC7055938; doi:10.1161/HYPERTENSIONAHA.119.13926)

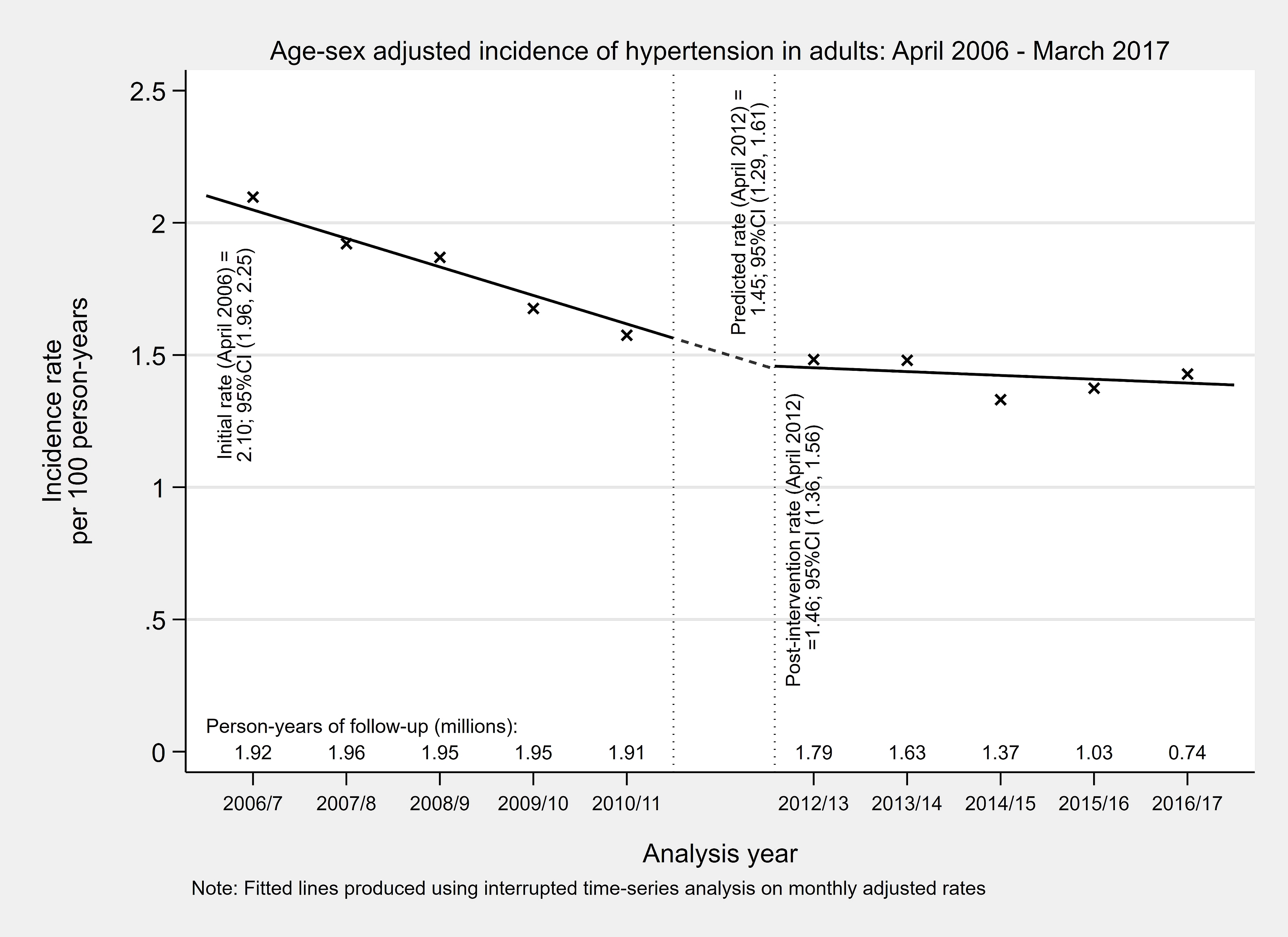

Supplement: Supplementary file 2 [file hyp-75-356-s002.jpg]
